# Cancers and erectile dysfunction: a Mendelian randomization study

**DOI:** 10.3389/fendo.2024.1417830

**Published:** 2024-11-06

**Authors:** Ge Yang, Yuanguo Xiong, Ziwen Wang, Jingsong Wang, Yongchuan Chen, Hong Zhang

**Affiliations:** ^1^ Department of Pharmacy, Renmin Hospital of Wuhan University, Wuhan, China; ^2^ Department of Cardiology and Nephrology, The 82nd Group Army Hospital of PLA (252 Hospital of PLA), Baoding, China; ^3^ Department of Urology, Institute of Urologic Disease, Renmin Hospital of Wuhan University, Wuhan, China; ^4^ Department of Pharmacy, First Affiliated Hospital of Army Medical University, Chongqing, China

**Keywords:** cancer, erectile dysfunction, mendelian randomization, causal inference, bidirectional causal effects

## Abstract

**Purpose:**

Cancer often coexists with erectile dysfunction, yet the causal relationship between them remains unclear. This study aims to investigate the causal link between tumors and ED through Mendelian randomization.

**Method:**

Data on 13 different cancers, including lung cancer, colorectal cancer, testicular cancer, lymphoma, esophageal cancer, pancreatic cancer, thyroid cancer, bladder cancer and brain cancer were collected from various databases. ED data, comprising 2,205 cases and 164,104 controls, were sourced from the FinnGen project. Primary methods for MR analysis included IVW, MR-Egger, weighted median, and weighted mode.

**Results:**

IVW results revealed associations between colorectal cancer (OR=1.17;95% CI 1.02-1.13, p=0.0252), prostate cancer (OR=1.63;95% CI 1.52-1.75, p<0.001) and liver cancer (OR=0.93;95% CI 0.88 -0.99, p=0.012) with ED.

**Conclusion:**

Mendelian randomization analysis supports that prostate cancer and colorectal cancer are associated with an increased risk of Erectile Dysfunction, whereas liver cancer is linked to a decreased risk of ED. No evidence suggests that ED contributes to an increased risk of prostate cancer.

## 1 Introduction

Erectile dysfunction (ED), characterized by the persistent inability to achieve or maintain an erection sufficient for satisfactory sexual performance, is a prevalent and profound male health concern. Epidemiological studies have indicated a significant global prevalence of ED, estimating that approximately 40% of men over the age of 40 experience varying degrees of ED. With advancing age, the incidence of ED escalates, rendering it an increasingly prevalent issue in the context of an aging population (1, 2). While ED may have multifactorial etiologies, encompassing psychological, vascular, and hormonal factors (3–5), its implications for the affected individuals’ quality of life and psychological well-being are undeniable. As cancer treatments continue to advance, resulting in improved survival rates among cancer patients, questions have arisen regarding the potential repercussions of these treatments on sexual health.

Notably, within the context of prostate cancer(Pca), surgical interventions, particularly prostatectomy, have been implicated in a significant risk of postoperative ED (6). Moreover, in instances of advanced or aggressive disease, Androgen Deprivation Therapy (ADT)—encompassing both surgical and medical castration—is frequently employed as a therapeutic modality. However, this treatment strategy not only amplifies the predisposition to ED but may also engender lasting sexual dysfunction (7). While the scholarly community acknowledges the correlations between cancer treatments and ED, the precise etiological relationship between the two remains elusive. Despite a prevailing focus on Pca within the ED-cancer interface, investigations into the association between ED and other malignancies have been conspicuously sparse. The complex dynamics and potential causal pathways connecting malignant tumors with sexual health remain insufficiently characterized.

Conducting randomized controlled trials to investigate the relationship between malignant tumors and ED is challenging and requires considerable human and material resources. In addition, traditional research may be plagued by confounding factors that are difficult to eliminate, and there may be causal reversal between exposure and outcome. Mendelian randomization (MR) is an epidemiological approach rooted in genetic epidemiology that uses data from genome-wide association studies (GWAS) to use genetic variants as instrumental variables to assess causal relationships between modifiable exposures and specific diseases (8). Since genetic variation is determined at conception and randomly assigned, this design is less prone to confounding factors and reverse causation bias compared to traditional observational studies, making it highly suitable for testing causal hypotheses. The aim of this study is to use MR, building on GWAS data, to test the existence of a causal association between cancers and ED.

## 2 Materials and methods

### 2.1 Study design

**Figure 1:**
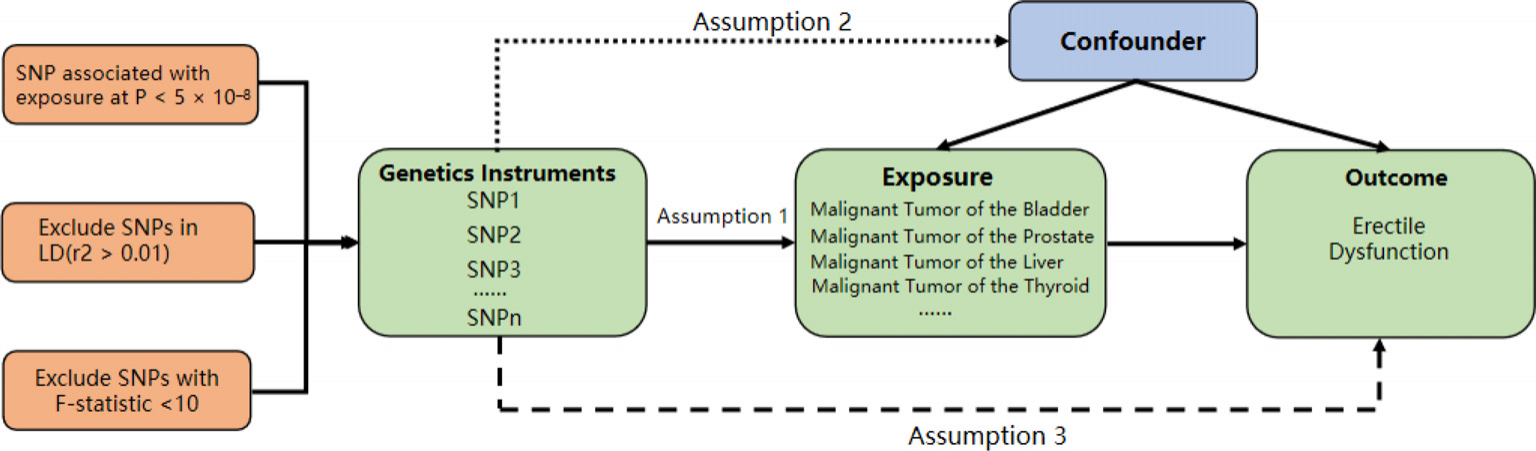
Study design for MR analysis. This figure illustrates the three key components of the mendelian randomization (MR) study: (1) Identification of genetic variants as instrumental variables for malignancies, (2) Acquisition of data summaries from GWAS, and (3) Harmonization of summary data for SNP effects on erectile dysfunction (ED) determined by the GWAS gene instrument.

The MR study design comprises three key components: (1) identification of genetic variants suitable as instrumental variables for malignancies, (2) acquisition of data summaries from GWAS with the purpose of genetic instrumenting, and (3) procurement and harmonization of summary data for Single Nucleotide Polymorphism (SNP) effects on ED, determined by the GWAS gene instrument (**Figure 1**). All studies used in the MR analysis had undergone ethical review and were approved by relevant ethical review boards and participants had provided informed consent prior to their data being used in the analysis.

### 2.2 Data sources

We acquired summary-level data on exposure from GWAS. In order to meet the assumption of homogeneity required for the two-sample Mendelian randomization analysis, individuals of non-European descent were entirely excluded. Our initial step involved conducting a two-sample Mendelian randomization using tumor data sourced from the Finnish database. The GWAS summary data for ED were extracted from FinnGen (https://r5.finngen.fi/), including 2205 patients and 164,104 controls of European ancestry. Subsequently, we conducted a reverse Mendelian randomization MR analysis to investigate the causal relationship between prostate cancer and ED. This approach allowed us to assess whether prostate cancer has a causal impact on ED, ensuring a bidirectional evaluation of the potential causal relationship between the two conditions.

### 2.4 Instrumental variable selection and data harmonization

In our MR analyses, we used SNPs as instrumental variables (IVs) to estimate the effect of tumor on ED. the selection of IVs was based on genome-wide significance, with a p-value threshold of less than 5 × 10^-6^. To ensure that independent instruments for mediation analyses were selected. We standardized the genetic variants according to the effects of the various studies. we considered only those variants for which genetic summary statistics were available for all traits examined in a given analysis. This approach ensured consistency in the instrumental variables used across analyses and did not use proxies to maintain the integrity of the results. For all of the categorized to maintain the consistency of the variables used as instrumental variables in the different analyses, no proxies were used. To assess instrumental strength, we calculated the f-statistic. We considered an f-statistic greater than 10 to be an indication of robust instrumental strength.

### 2.5 Statistical analysis

Statistical analysis in our study, we used the inverse variance weighting (IVW) method as the primary method for univariate analysis. This technique combines results obtained from a single snp using a random effects meta-analysis approach. To ensure consistency in causal direction, we conducted additional analyses using other methods: MR Egger and Weighted median. To assess heterogeneity among the included SNPs, we used Cochran’s Q statistic in both IVW and MR-Egger models. p-values below 0.05 were considered significant for heterogeneity. We also used MR-Egger regression to explore potential pleiotropy, which refers to situations in which genetic variation affects multiple traits. To address any potential problems associated with pleiotropy, we used MR-PRESSO, which allowed us to identify and remove potential outliers that could introduce potential pleiotropy and bias our results.

## 3 Result

### 3.1 Study overview

We identified a significant number of independent SNPs linked to following traits (P < 5×10^-6^): 61 SNPs were linked to the prostatic cancer, 21 SNPs were linked to the bladder cancer, 8 SNPs were linked to the nonseminomatous testicular cancer, 25 SNPs were linked to the lung cancer, 25 SNPs were linked to the thyroid cancer, 27 SNPs were linked to the colorectal cancer, 6 SNPs were linked to the spermatogonial testicular cancer, 8 SNPs were linked to the malignant tumors of the brain, 6 SNPs were linked to the esophagus cancer, 14 SNPs were linked to the gastric cancer, 16 SNPs were linked to the pancreatic cancer, 25 SNPs were linked to the hematologic neoplasms. Additionally, we also excluded confounder-related SNPs, outcome-related SNPs, and outliers SNPs before MR analysis.

### 3.2 The causal effect of cancer on ED

**Figure 2:**
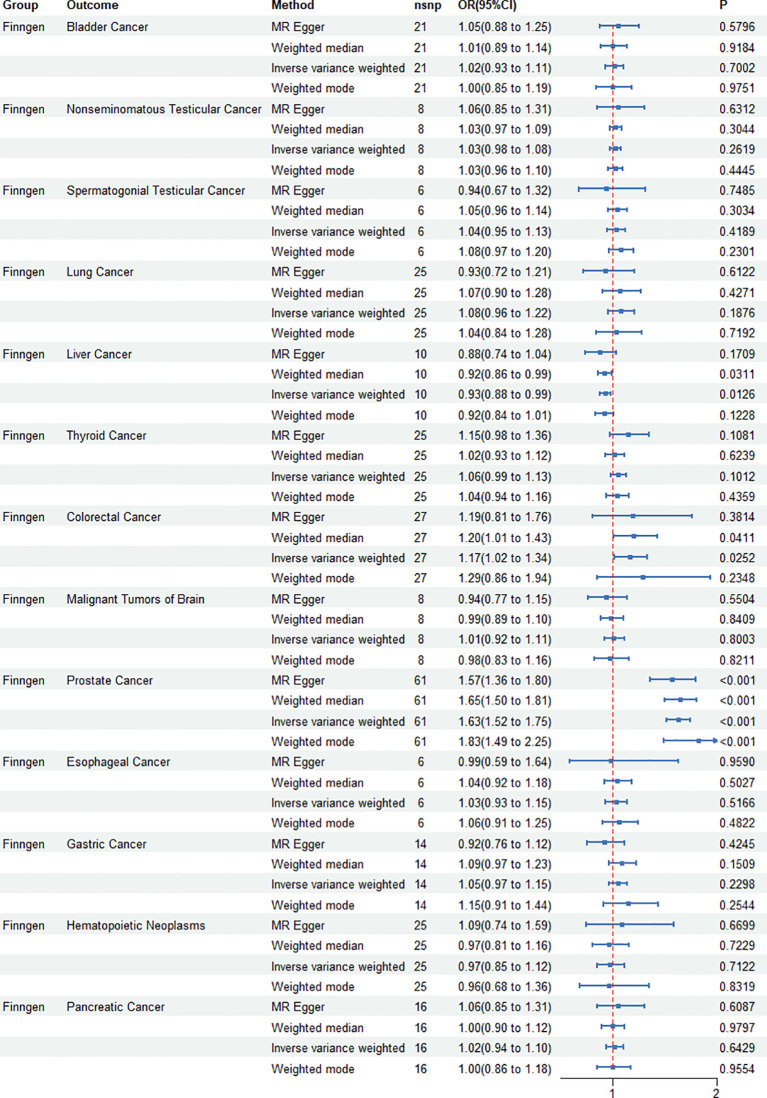
Causal effect of genetically predicted cancers on erectile dysfunction (ED). Four mendelian randomization (MR) methods—IVW, MR-Egger, weighted median, and weighted mode—are used to analyze the impact of various cancers, including prostate and colorectal cancers, on the risk of ED. The associations are visualized using odds ratios and confidence intervals for each cancer type.

IVW, MR–Egger, weighted median, and weighted mode regression analyses were undertaken to examine the causal impact of genetically predicted cancers on ED (see **Figure 2**). Our results showed that the causal effect of cancer on ED, as shown by IVW, Pca was significantly associated with increased risk of ED (IVW odds ratio OR=1.63;95%CI 1.52 – 1.75, p<0.001), and colorectal cancer was also significantly associated with increased risk of ED (IVW odds ratio OR=1.17;95%CI 1.02–1.34, p=0.0252). Liver cancer was significantly associated with decreased risk of ED (OR=0.93;95%CI 0.88–0.99, p=0.012). The MR power in the present study was calculated at 83% using the “mRnd” tool.

### 3.3 The causal effect of ED on prostatic cancer

**Figure 3:**
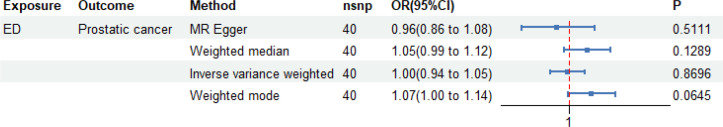
Causal effect of genetically predicted erectile dysfunction (ED) on prostate cancer. The mendelian randomization (MR) study findings indicate that genetic predisposition to ED does not alter the risk of developing prostate cancer. Four MR methods—IVW, MR-Egger, weighted median, and weighted mode—are used to assess potential causal relationships.

IVW, MR–Egger, weighted median, and weighted mode regression analyses were conducted to assess the causal effect of genetically predicted ED on prostate cancer (**Figure 3**). The MR study found that the genetic predisposition to ED did not change the risk of developing prostate cancer.

### 3.4 Sensitivity analysis

Cochrane’s Q and Egger’s regression equations were employed to assess heterogeneity and horizontal pleiotropy. When examining the causal relationship between Pca and ED, heterogeneity was observed (p = 0.019). Likewise, in the investigation of the causal relationship between colorectal cancer and ED, heterogeneity was detected (p = 0.037). Our results showed the presence of horizontal pleiotropy in both prostate and colorectal cancers, as evidenced by statistically significant p values (<0.05) obtained using the MR-PRESSO approach. However, this phenomenon was not observed when the MR-Egger method was used.

## 4 Discussion

The sexual function of male cancer patients is influenced by various factors, including age, medications, anxiety, frailty, and other variables (2, 9–11). Despite the extensiveness of research on survival duration among cancer patients, the broader aspect of their overall quality of life, particularly their sexual well-being, has received limited attention. To address this knowledge gap, we employed a Mendelian randomization strategy using two independent samples to systematically investigate the potential associations between various malignancies and ED. Our analysis revealed a consistent and robust association between Pca and colorectal cancer with an increased risk of ED—a finding replicated across distinct analytical models. This observation not only reinforces the validity of our conclusions but also highlights the importance of considering the impact of specific tumor types on sexual function in male cancer patients.

Substantial variability exists in the reported rates of ED among cancer patients in various studies, potentially attributed to factors such as cancer staging, site, type, surgical procedures, and treatment medications. In the clinical setting, the assessment of sexual function in male cancer patients before and during treatment is often based on patient self-reporting. However, this reliance can be compromised by factors such as limited self-awareness, feelings of shame, or cultural norms fostering reticence, which may lead to distorted representations of patients’ sexual performance and experiences. A cross-sectional and meta-analysis encompassing 43 studies and 13,148 participants established an overall ED prevalence of 40.72% (95%CI 31.80-50.29) among cancer patients. Notably, the prevalence was found to be 28.60% (95% CI 12.10-53.83) at the time of diagnosis and escalated to 42.70% (95% CI32.97-53.03) following treatment, indicating marked fluctuations across different disease stages and cancer types (12). A separate systematic review and meta-analysis further demonstrated disparate ED rates among patients diagnosed with bladder, colon, prostate, testicular, and rectal cancers. During treatment, the overall prevalence stood at 13% (95% CI 7-19), with the highest incidence (45%) observed in patients undergoing surgery and radiation therapy, and the lowest (11%) reported in those treated with surgery and chemotherapy. Among treated patients, retrograde ejaculation was prevalent in 45% (95% CI 24-65). Post-treatment, bladder cancer survivors universally experienced dry orgasms, whereas Pca survivors displayed the highest ED prevalence at 57% (13). In Southeast Asia, a cross-sectional study on lymphoma survivors identified fatigue as the predominant cause of sexual inactivity, affecting 81.7% of whom experienced varying levels of ED. Among younger patients, 64.5% reported ED, and after adjusting for confounders, age emerged as the sole statistically significant correlate (p < 0.005), with ED severity escalating with advancing years (14). Another cross-sectional investigation revealed that 58% of young and middle-aged cancer patients exhibited some degree of ED (15). Moreover, a survey concentrating on bladder cancer patients disclosed an 80% prevalence of ED, with a more substantial decline in sexual function subsequent to radical surgery (16). In summary, eliminating all potential confounders in clinical research remains a formidable challenge. Consequently, we employed MR as a causal analytical tool to mitigate the influence of potential confounding factors.

Our findings reveal a causal association between Pca and ED, aligning with contemporary clinical research outcomes (6, 17–23). Globally, healthcare systems are endeavoring to safeguard the sexual function of Pca patients. They employ minimally invasive procedures, radiation, and other techniques with minimal impact to prevent harm to nerves and vital tissues associated with sexual function. Regrettably, fewer than 30% of Pca patients undergo baseline assessment of male sexual function before commencing Pca treatment (24). Conversely, elderly Pca patients may encounter challenges in responding to inquiries about sexual matters due to the effects of aging, abnormal anxiety, and pain. The association between Pca and ED appears to be underpinned by biological mechanisms. Similar to benign prostatic hyperplasia, Pca can induce changes in the lower urinary tract, subsequently affecting sexual activity (25). Pca itself secretes various inflammatory factors, causing damage to vascular endothelium—an established primary cause of ED (26). Moreover, ADT could inhibit sexual function, while Pca surgery may also result in injury to nerves involved in erectile function.

The significant association observed between colorectal cancer and ED in our study sheds light on an important aspect of cancer-related complications. Our findings suggest that individuals genetically predisposed to colorectal cancer are at an increased risk of experiencing ED, as indicated by the statistically significant association revealed by the IVW analysis (OR = 1.17, 95% CI 1.02 – 1.34, p = 0.0252). In contrast, our study revealed an unexpected finding regarding the relationship between liver cancer and ED. Contrary to expectations, we observed a significant association between liver cancer and a decreased risk of ED. The IVW analysis demonstrated an inverse association, suggesting a protective effect of liver cancer against the development of ED (OR = 0.93, 95% CI 0.88 – 0.99, p = 0.012). The incidence of hepatocellular carcinoma is notably higher in men than in women (27), suggesting a potential pivotal role of estrogen in the protective dynamics against hepatocellular carcinoma. Nonetheless, it is essential to acknowledge that estrogen is also converted to testosterone in men. Testosterone and free testosterone are further modulated by sex hormone-binding proteins and plasma albumin. Disturbances in liver cell function significantly influence plasma levels of sex hormone-binding proteins and plasma albumin, consequently impacting the free testosterone/estradiol ratio. This altered ratio, in turn, influences the expression of inflammatory factors in the vascular endothelium (22, 23). Notably, male patients with hepatocellular carcinoma exhibit a lower free testosterone/estradiol ratio compared to their normal counterparts (28). Elevated estradiol levels may play a vital role in safeguarding the vascular endothelium. However, it is important to recognize that excessively high estrogen levels may, conversely, contribute to diminished sexual function in men. In conclusion, hepatocellular carcinoma may potentially exert a protective influence on ED through sex hormones, yet additional evidence elucidating its underlying mechanisms is imperative.

Our research indicates no clear link between testicular cancer and ED. Those with testicular cancer are usually younger and face fewer age-related ED risk factors (29). Treatment approaches for testicular cancer, such as unilateral testicular resection surgery, and psychological aspects such as body image perception, play crucial roles in impacting sexual function (30). Studies propose a higher short-term risk of ED development in testicular cancer patients compared to the long term, likely due to treatment-related psychological stress and physical trauma, with potential for ED conditions to improve in extended follow-up (31, 32). Longitudinal study data indicates that in a follow-up of approximately 7.5 years involving 143 Caucasian-European testicular cancer survivors, the median time to erectile function recovery was 60 months for those who received radiotherapy, 60 months for those who underwent chemotherapy, and 70 months for those who underwent retroperitoneal lymph node dissection (33). This improvement could be attributed to the recovery and adaptation of the body, as well as psychological adaptation and coping strategies.

This MR study, investigating the relationship between cancer and the risk of ED, represents the inaugural exploration of its kind. Our approach involved selecting SNPs that are strongly correlated, independently inherited, and unrelated to linkage disequilibrium as instruments to estimate the causal impact of cancer on ED risk. The use of F-statistics well above 10 to gauge the strength of the instruments’ connection with cancer indicates a low likelihood of bias stemming from weak instruments. Through the employment of robust tools (F > 10), we minimized the potential bias arising from sample overlap. Furthermore, the MR analysis uncovered the genetic influence of cancer on ED risk, overcoming the limitations associated with observational studies susceptible to confounding factors. Nonetheless, it is essential to recognize the limitations of our study. Firstly, the summary data from GWAS only pertain to individuals of European descent, necessitating caution in generalizing our findings to racially and ethnically diverse populations. Secondly, due to the unclear exact functions of some SNPs in the instruments, residual bias may exist.

## 5 Conclusion

Our study has found a causal association between Pca, colorectal cancer, liver cancer and ED. Pca and colorectal cancer increase the risk of ED, whereas liver cancer decreases this risk. However, further investigations are needed to elucidate the underlying mechanisms governing the causal association between tumors and ED.

## Data Availability

The original contributions presented in the study are included in the article/**Supplementary Material**, further inquiries can be directed to the corresponding author.
