# Supplementary figures and images for: Cancers and erectile dysfunction: a Mendelian randomization study
Source: Front Endocrinol (Lausanne). 2024 Nov 6;15:1417830. doi: 10.3389/fendo.2024.1417830 (PMC11578273; doi:10.3389/fendo.2024.1417830)

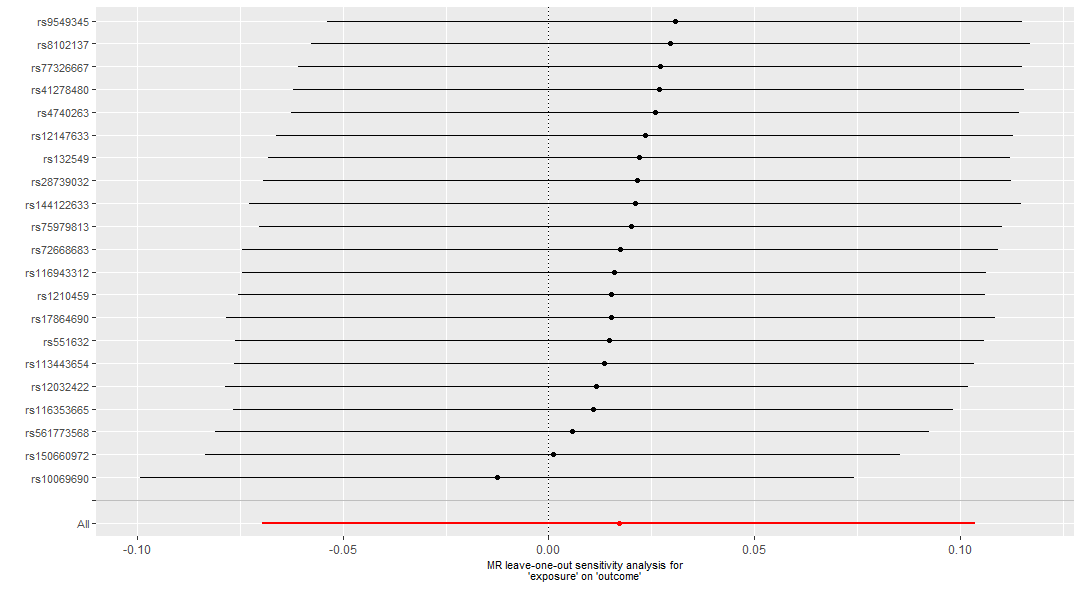

Supplement: Supplementary file 1 [file Image1.tiff]

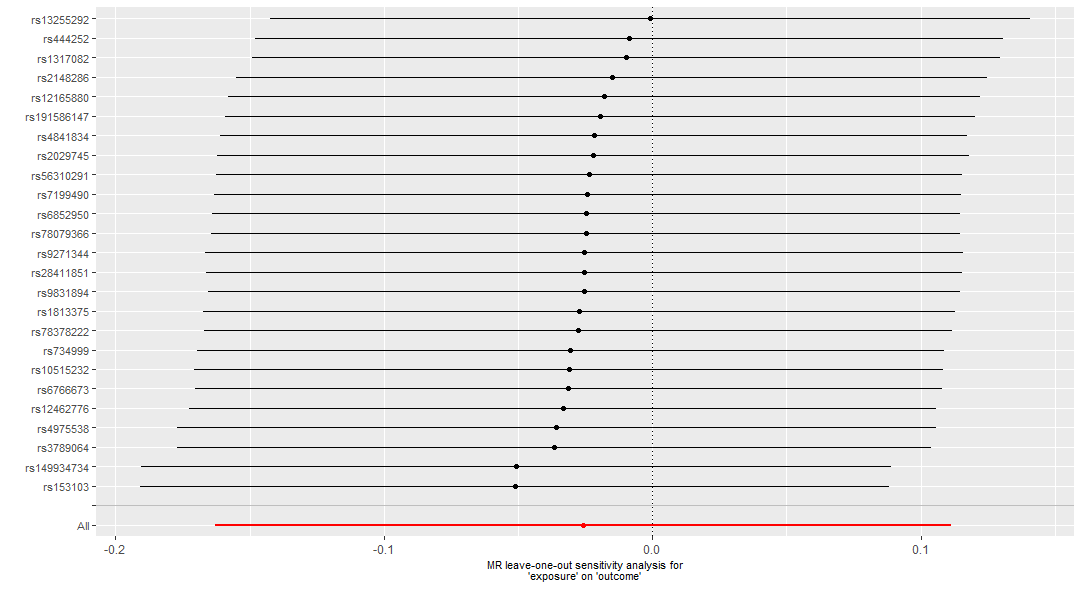

Supplement: Supplementary file 2 [file Image2.tiff]

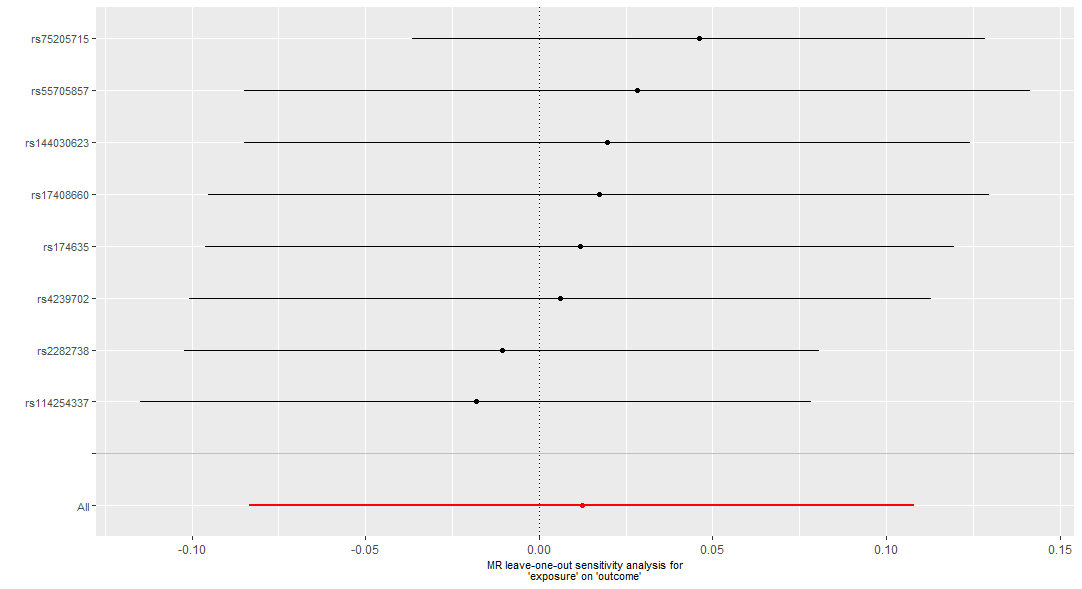

Supplement: Supplementary file 3 [file Image3.tiff]

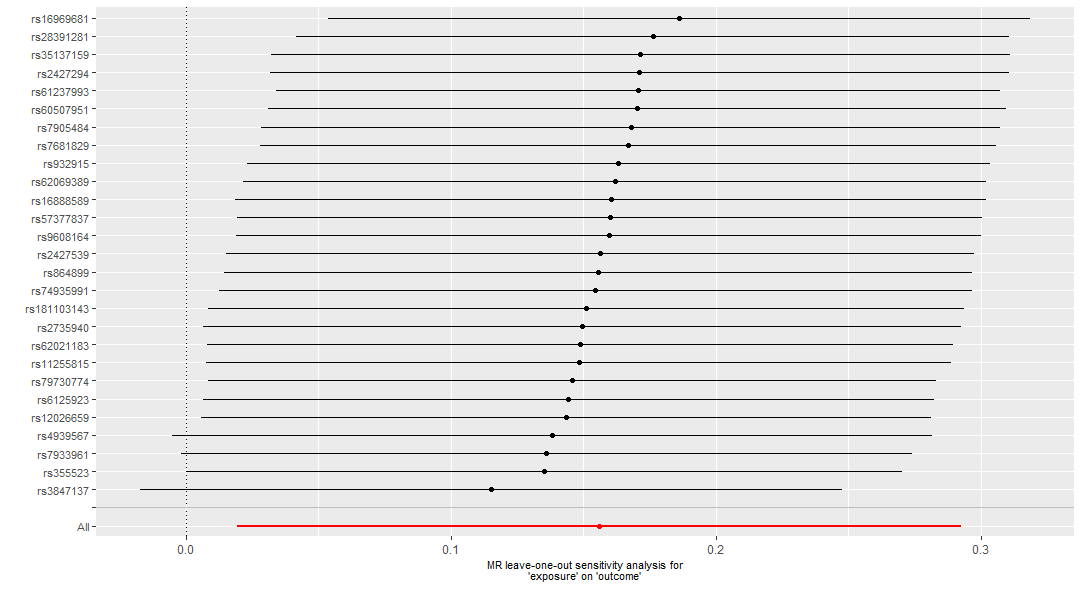

Supplement: Supplementary file 4 [file Image4.tiff]

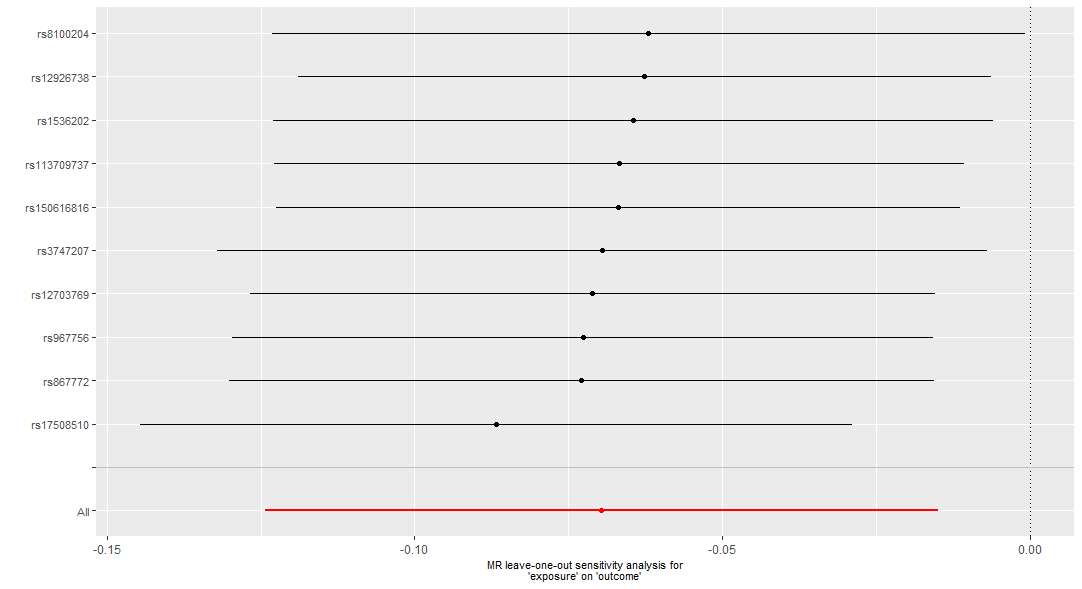

Supplement: Supplementary file 5 [file Image5.tiff]

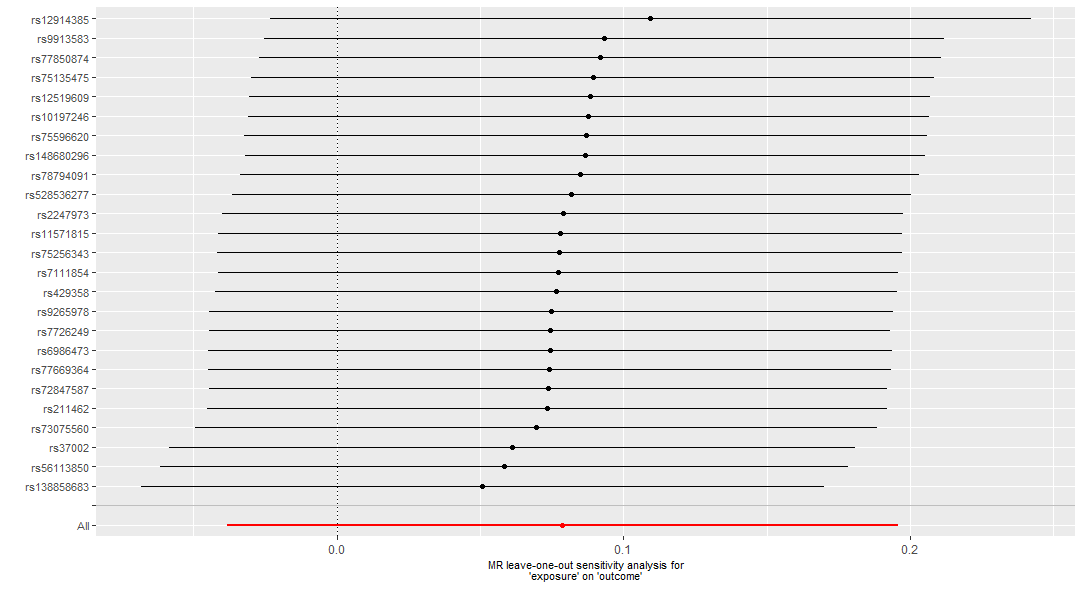

Supplement: Supplementary file 6 [file Image6.tiff]

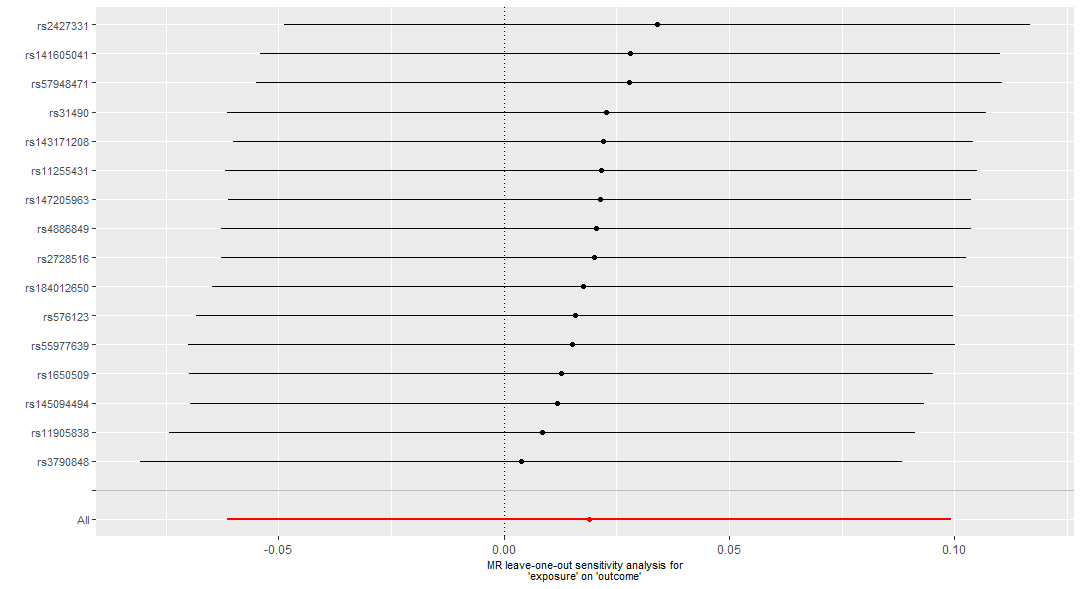

Supplement: Supplementary file 7 [file Image7.tiff]

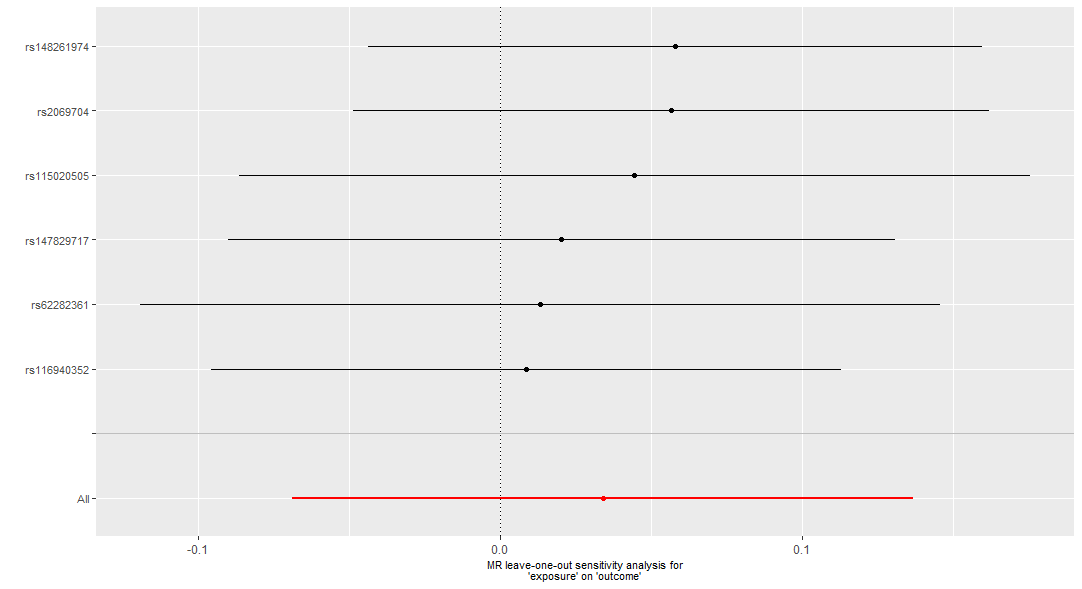

Supplement: Supplementary file 8 [file Image8.tiff]

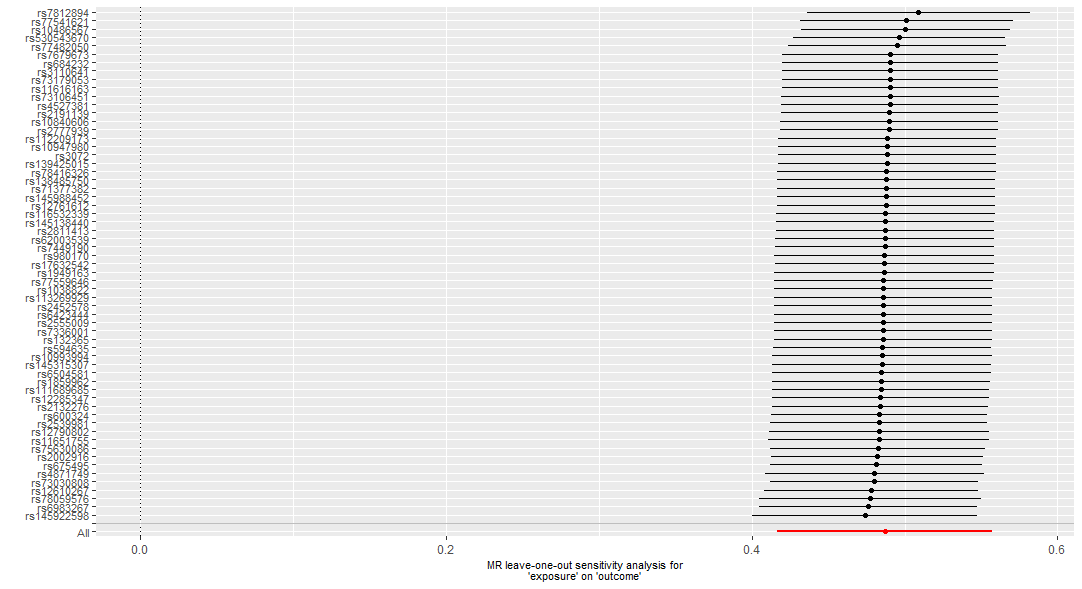

Supplement: Supplementary file 10 [file Image10.tiff]

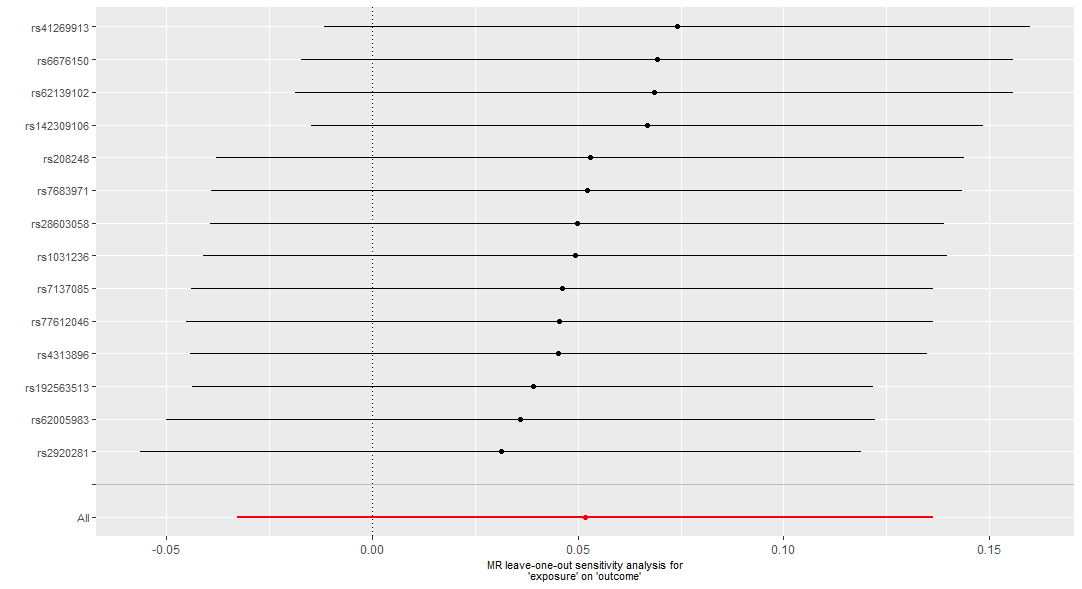

Supplement: Supplementary file 11 [file Image11.tiff]

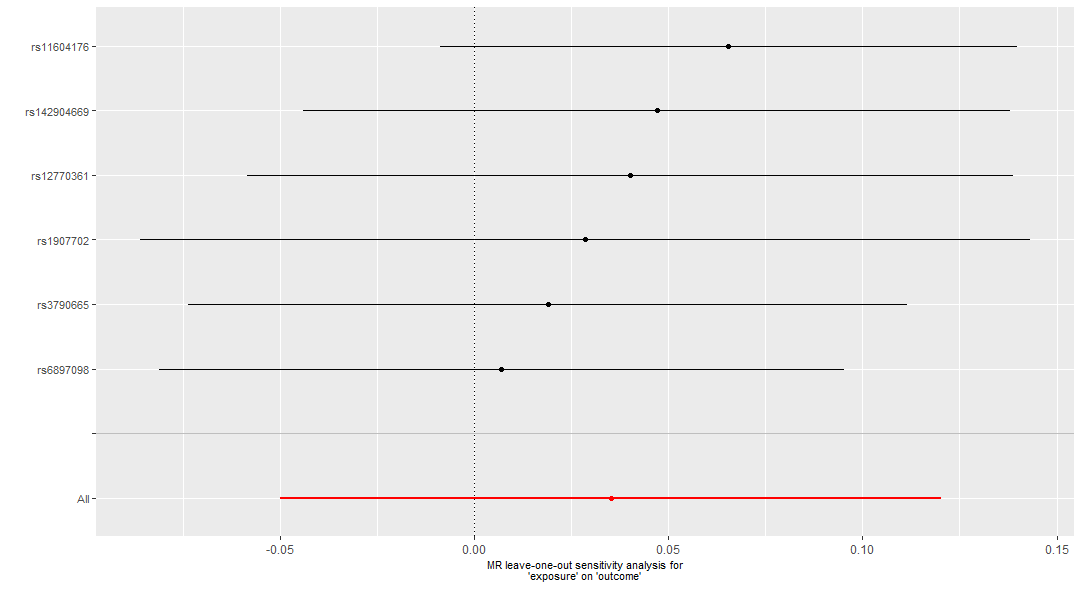

Supplement: Supplementary file 12 [file Image12.tiff]

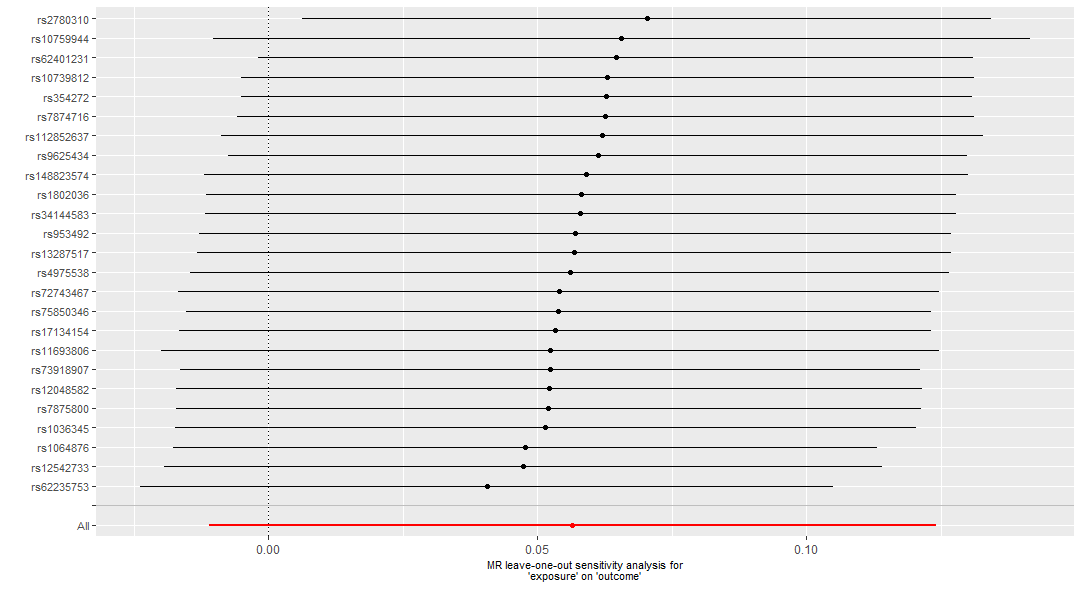

Supplement: Supplementary file 13 [file Image13.tiff]
